# Post-Fire Coleoptera Fauna in Central Russian Forests after the 2021 Fires (Study Using Beer Traps)

**DOI:** 10.3390/insects15060420

**Published:** 2024-06-05

**Authors:** Leonid V. Egorov, Alexander B. Ruchin, Alexander I. Fayzulin

**Affiliations:** 1 Joint Directorate of the Mordovia State Nature Reserve and National Park ”Smolny”, Saransk 430005, Russia; ruchin.alexander@gmail.com (L.V.E.); platyscelis@mail.ru (A.B.R.); 2 Prisursky State Nature Reserve, Cheboksary 428034, Russia; 3 Institute of Ecology of Volga River basin of RAS, Samara Federal Research Center of RAS, Togliatti 445003, Russia

**Keywords:** insects, species composition, wildfire, Coleoptera, Republic of Mordovia

## Abstract

**Simple Summary:**

This study presents an analysis of the Coleoptera fauna in the forests of the Euro-pean Russia in 2022 and 2023, after a fire. Insects were sampled from burned plots (9) in 2010 and 2021, as well as unburned (control) plots (2), and alpha diversity was compared. After processing the material, we examined a total of 12,218 Coleoptera specimens from 38 families and identified 194 species. The families Nitidulidae, Cerambycidae, Elateridae, and Scarabaeidae were the most abundant across all plots. Cerambycidae, Elateridae, Nitidulidae, Staphylinidae, Coccinellidae, and Scarabaeidae exhibited the greatest species diversity. In total, 17 species were found on all plots. Only five species exhibited preferences for some certain plots. Maximum abundance and species diversity were observed on unburned (control) plots. The plots where fires occurred in 2010 and 2021 had the lowest total abundance values for Coleoptera. These fires destroyed almost all potential sites for beetle settlement, feeding, breeding, and shelter.

**Abstract:**

Fires can significantly impact forest ecosystems. However, studies on the effects of fires on insect communities in post-fire plots in natural forests are rare. This study presents an analysis of the Coleoptera fauna in the forests of the Mordovia State Nature Reserve (European Russia) in 2022 and 2023 after a fire. Insects were sampled from burned plots (9) in 2010 and 2021, as well as unburned (control) plots (2), and alpha diversity was compared. After processing the material, we examined a total of 12,218 Coleoptera specimens from 38 families and identified 194 species. The families Nitidulidae, Cerambycidae, Elateridae, and Scarabaeidae were the most abundant across all plots. Cerambycidae, Elateridae, Nitidulidae, Staphylinidae, Coccinellidae, and Scarabaeidae exhibited the greatest species diversity. In total, 17 species were found on all plots, including *Cetonia aurata*, *Protaetia cuprea volhyniensis*, *Trogoderma glabrum*, *Carpophilus hemipterus*, *Epuraea biguttata*, *Glischrochilus grandis*, *Glischrochilus hortensis*, *Glischrochilus quadripunctatus*, *Soronia grisea*, *Pediacus depressus*, *Chrysanthia geniculata*, *Anastrangalia reyi*, *Leptura quadrifasciata*, *Leptura thoracica*, *Lepturalia nigripes*, *Rhagium mordax*, and *Anisandrus dispar*. Only five species exhibited preferences for certain plots. Maximum abundance and species diversity were observed on unburned (control) plots. The plots where fires occurred in 2010 and 2021 had the lowest total abundance values for Coleoptera. These fires destroyed almost all potential sites for beetle settlement, feeding, breeding, and shelter. Traps recorded a higher abundance of Coleoptera in the first year after fires compared to the second year. The Coleoptera fauna showed the greatest similarity on the control plots.

## 1. Introduction

In forest ecosystems, Coleoptera are a significant community component that plays a crucial role in substance cycling and redistribution, organic matter decomposition, pollination, and biological control [1,2,3,4,5]. Most publications that discuss the effects of wildfires on Coleoptera fauna focus on soil-dwelling and terrestrial Coleoptera, which are convenient as monitoring objects [6,7,8,9,10,11,12]. Large fires can have both direct and indirect effects on Coleoptera. Many species of Coleoptera have been observed attempting to escape the flames or burrowing into moist soil to avoid fire [13,14,15,16,17]. The effects of fire are directly influenced by its size, speed of development, and catastrophic nature, as well as the ecological conditions of the habitat. In addition to the direct effects, there are also indirect effects that may not be immediately apparent but can occur gradually over time [18,19,20].

Concerning the response to fires varies among Coleoptera species, some species thrive immediately after a fire, even if they were not previously recorded in the habitats where the fires occurred. Other species gradually repopulate burned ecosystems as they adapt to the effects of fires. A third group return to burned areas after previously inhabiting them. Coleoptera species are more susceptible to fire during their egg and larval stages due to their limited mobility and specific habitat requirements [9,21,22,23,24,25].

The analysis of climatic indicators in European Russia from 1966 to 2018 indicates that the air temperature increased at a rate of 0.32–0.53 °C per decade. The number of days per year with average daily air temperature above 10 °C also increased. These data indicate a significant warming of the climate, which creates conditions for an increase in the danger of wildfires in various regions of Russia [26,27]. According to historical records and palynological analysis, fires occurred in the Mordovia State Nature Reserve in 1842, 1899, 1932, 1972, 2010, 2018, 2019, and 2021. They differed significantly from each other in intensity, area, character of development, duration, and damage to the ecosystem [10,19,28,29]. The most significant wildfires were recorded during the summers of 2010 and 2021. In 2010, the fires took place in natural forests that were considered climax ecosystems at the time. Following the fires, changes occurred in the ecosystem of the burned areas, resulting in the emergence of young deciduous trees, primarily birch (*Betula*) and aspen (*Populus tremula*), and the restoration of the herbaceous layer. Khapugin et al. [30] reported that dead and weakened trees gradually decomposed at the site of the upstream fires after 2010. This resulted in a rise in dry wood, primarily from coniferous species that had not had time to decompose. Since 2017, there has been a gradual decrease in precipitation, soil moisture, and changes in the hydrological regime of surface water. The catastrophic fires of 2021 that occurred in the same areas burned in 2010 were caused by a variety of factors. In many places, the fires burned deadwood and dry trees [31]. The situation that occurred in the forest ecosystem of the Mordovia State Nature Reserve is unique to this location. Therefore, we conducted research in this area in 2022 and 2023. The aim of this work was to study the Coleoptera fauna collected by beer traps in the forests of the Mordovia State Nature Reserve affected by wildfires in 2021. The objectives of the study were as follows: (1) to analyze the biodiversity and abundance of Coleoptera on both burned and unburned plots; (2) to compare the number of saproxylic and anthophilic species on the studied plots; and (3) to compare the similarity of Coleoptera faunas in burned and unburned plots and to find out the possibilities of species occupation of the burned plots.

## 2. Materials and Methods

### 2.1. Study Area

This study was conducted from 2022 to 2023 in the Mordovia State Nature Reserve in center of European Russia. The reserve is situated on the southern boundary of the taiga zone and adjacent to forest-steppe ecosystems. The Mordovia State Nature Reserve has been under state protection since 1936. The protected area comprises mainly forest ecosystems and covers a total area of 321.62 km^2^. Historically, the primary tree species in this area was a pine (*Pinus sylvestris* L.), which formed pure or mixed forest communities. However, due to the 2010 fires, many pine forests were replaced by birch (*Betula pendula* Roth), resulting in deformations. The Mordovia State Nature Reserve contains pure forest communities of linden (*Tilia cordata* Mill.) in the northern region. Oak forests (*Quercus robur* L.) are found in smaller areas, mainly in the floodplain of the Moksha River in the western part of the reserve. The soils in this area are predominantly sandy, with varying degrees of podzolization. The average annual precipitation ranges from 406.6 to 681.3 mm, while the average annual air temperature is 4.7 °C. Maximum values are recorded in July (+39–40 °C) and minimum values in February (−42–45 °C) [30].

### 2.2. Sampling Procedures

**Figure 1:**
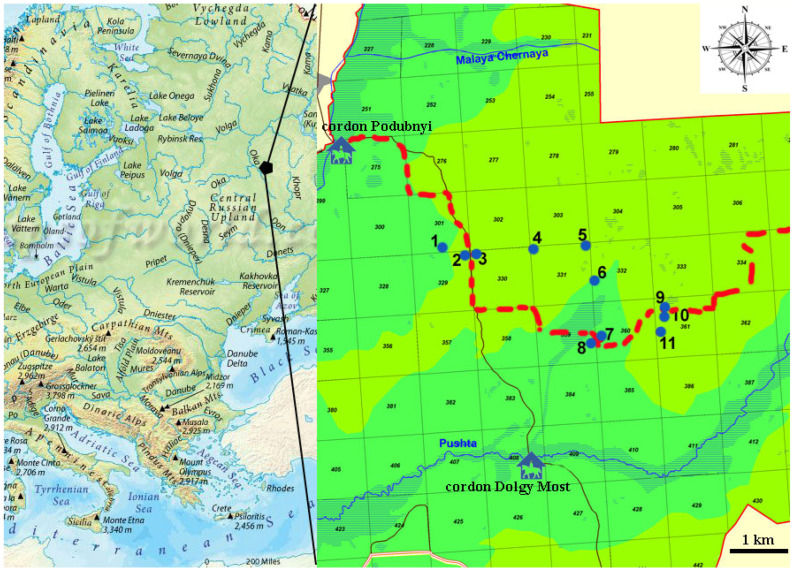
Geographical position of the Mordovia State Nature Reserve in Europe. Study plots are named according to designations in the text. The red line shows the boundary of the fires of 2021 (fires were north of this line). Darker green areas are lower elevations and lighter green higher elevations. A brief description of the plots1–11 is given in Table 1.

Material was collected between April and October using beer traps baited with beer and sugar. Each trap was made from a 5 L plastic container that previously contained water. A 10 × 10 cm square hole was cut out on one side of the container. It is necessary for the arrival of beetles. Bait made of beer and sugar was poured into the bottom of the container [32]. One trap was set per plot, with the trap set at a height of 1.5 m, hanging below a small wooden tripod [33]. Traps were checked depending on weather conditions after 7–14 days. At the same time, after each check, all the bait was poured out and a completely new bait was added. Material was sampled 13 times in 2022 and 15 times in 2023 and then summarized. A total of 11 plots were selected for the study (Figure 1). These plots varied in terms of fire intensity, distance from the fire edge, and degree of vegetation recovery following the 2010 fires. Previously, Ruchin [31] published detailed descriptions and photos of all plots. We sampled plots that burned in 2010 and 2021 (plots 3, 4, 5, 6, 7, and 9), plots that burned only in 2010 (plots 1, 2, and 8), and control plots (plots 10 and 11) that never burned. A brief comparative description of plots is provided in Table 1.

### 2.3. Identification

The nomenclature of Coleoptera was determined using the latest catalogue of Palaearctic Coleoptera [34,35,36,37,38,39,40,41,42] and other relevant publications. Species within each family were listed based on modern data [43,44]. The years of description for some beetle species were determined according to Bousquet [45]. Species identification was performed by the L.V. Egorov.

### 2.4. Data Analyzes

The Jaccard index was used for comparison based on all beetle species identified on different plots. Additionally, we calculated the Margalef, Shannon, and Simpson indexes [46,47,48] to understand species diversity and community equitability. In our calculations, we excluded Coleoptera that were not identified at the species level. Jaccard index similarity is presented as a dendrogram built using Ward’s method and Ecuclidian distance. We determined saproxylic species based on guidelines from publications [49,50,51,52] and our own data [53]. Anthophilous species were defined as those that repeatedly visited flowers. Our own long-term observations, as well as information from publications [33,54], were used in this case. For saproxylic and anthophilic species, calculations were made for the ratio of the number of these species to the total number of species in a particular plot and expressed as a percentage.

Statistical analysis was performed with PAST 4.07 [55]. The ordination techniques, using principal component analysis (PCA), defined the main gradients in the distribution of the studied species selected for analysis (we analyzed the species that were common to all plots). To provide an ecological interpretation of the ordination axes, studied sites based on the registered species abundance were plotted on the PCA ordination diagram as additional environmental data.

## 3. Results

In total, 12,218 Coleoptera specimens were identified. We identified 194 species from 38 families (Appendix A). Some specimens (332, 2.7%) could not be identified to the species level. The families Nitidulidae (3781 specimens, 30.9%), Cerambycidae (3223, 26.4%), Elateridae (2572, 21.1%), and Scarabaeidae (1027, 8.4%) had the highest total abundance across all plots in both years of the study. Cerambycidae (33 species, 16.8%), Elateridae (21, 10.7%), Nitidulidae (19, 9.6%), Staphylinidae (15, 7.6%), Coccinellidae (12, 6.1%), and Scarabaeidae (8, 4.1%) accounted for 86.8% of all captured specimens.

**Figure 2:**
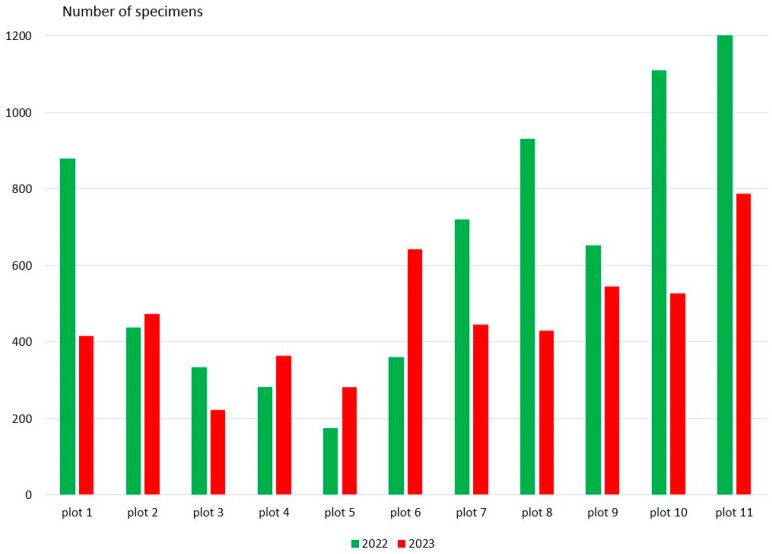
Coleoptera numbers on plots during by years of study.

The number of Coleoptera collected in traps varied among plots. The highest abundance of Coleoptera was observed on plot 11, plot 10, plot 8, and plot 1 (Figure 2). The plots with the lowest abundance values were plot 5, plot 3, and plot 4. Variations in Coleoptera abundance were observed across different years. In 2022, plots near the 2021 fire boundary (7, 8, 9, 10) and control plots (1, 11) had the highest beetle abundance. However, on plots 4, 5, and 6, which were far away from unburned forests and experienced intensive fires, beetle abundance was higher in 2023, the second year after the 2021 fire (Figure 2).

Plots 3, 4, and 5 had fewer beetle species than the rest of the plots, the latter of which ranged from 20 to 24 species each (Table 2). The diversity of Coleoptera species was highest on plots that either did not burn or burned only in 2010 in both years of the study. The lowest species diversity was observed on plots that burned in both 2010 and 2021 and were located deep within these fires.

The Shannon index yielded the highest value for plot 8. Overall, the burned plots from 2021 exhibited relatively high values for the Shannon index and low values for the Simpson index. The number of beetle species on these plots (4, 5, and 6) was significantly lower than on unburned plots (10 and 11), as evidenced by the low values of the Margalef index. The number of species was highest on plots 10 and 11. In contrast, plots bordering burned wood (7, 8, 9, 10, and 11) or unburned plots (10 and 11) had high Margalef index values. Meanwhile, unburned plots (10 and 11) had low Shannon index values and high Simpson index values, indicating the dominance of one or more Coleoptera species on these plots (Table 2).

**Figure 3:**
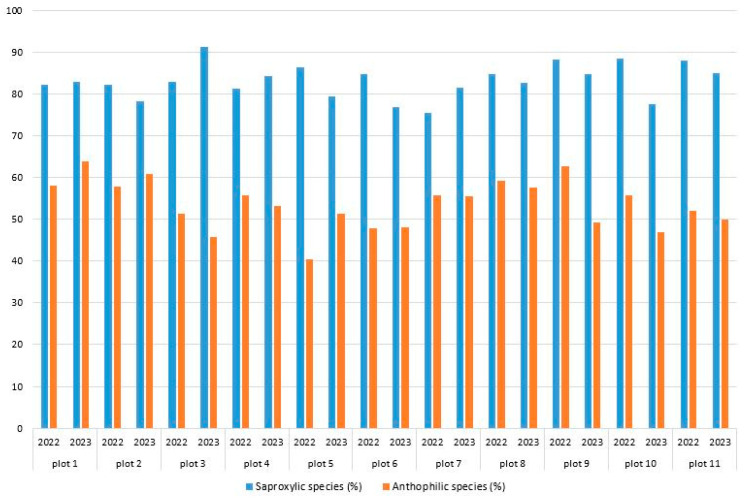
Percentages of saproxylic and anthophilic Coleoptera species on plots by year of study.

The percentage of saproxylic species varied among the plots studied, ranging from 75.8% (plot 6) to 87.0% (plot 9). Plot 6 had the lowest relative number of saproxylic species, while plots 9 and 3 (located on the boundary of burned plots) had the highest values (Table 2). There was no difference observed in the dynamics of the number of saproxylic species on individual plots during the study years. The fluctuations of this parameter on all plots varied by 10%. However, the parameter underwent the least amount of change on the unburned plots (1, 8, 10, 11), while the highest limit of variation was obtained on the plots burned in 2021 (Figure 3).

The percentage of anthophilic species varied among the studied plots, ranging from 45.5% (plot 6) to 59.7% (plot 1). Plot 6 had the lowest number of anthophiles, while plot 1 had the highest (Table 2). No changes in the number of anthophilic species by years of study were recorded on different plots. Somewhat larger changes of the parameter were obtained on boundary plots (Figure 3).

Out of the 194 species, 17 (8.6%) were present in all plots studied. The following are the taxa from the following seven families: (1) Scarabaeidae, *Cetonia aurata* (Linnaeus, 1758), *Protaetia cuprea volhyniensis* (Gory and Percheron, 1833); (2) Dermestidae, *Trogoderma glabrum* (Herbst, 1783); (3) Nitidulidae, *Carpophilus hemipterus* (Linnaeus, 1758), *Epuraea biguttata* (Thunberg, 1784), *Glischrochilus grandis* (Tournier, 1872), *Glischrochilus hortensis* (Geoffroy, 1785), *Glischrochilus quadripunctatus* (Linnaeus, 1758), *Soronia grisea* (Linnaeus, 1758); (4) Cucujidae, *Pediacus depressus* (Herbst, 1797); (5) Oedemeridae, *Chrysanthia geniculata* W.L.E. Schmidt, 1846); (6) Cerambycidae, *Anastrangalia reyi* (Heyden, 1889), *Leptura quadrifasciata* Linnaeus, 1758, *Leptura thoracica* Creutzer, 1799, *Lepturalia nigripes* (De Geer, 1775), *Rhagium mordax* (De Geer, 1775); (7) Curculionidae, *Anisandrus dispar* (Fabricius, 1792).

**Figure 4:**
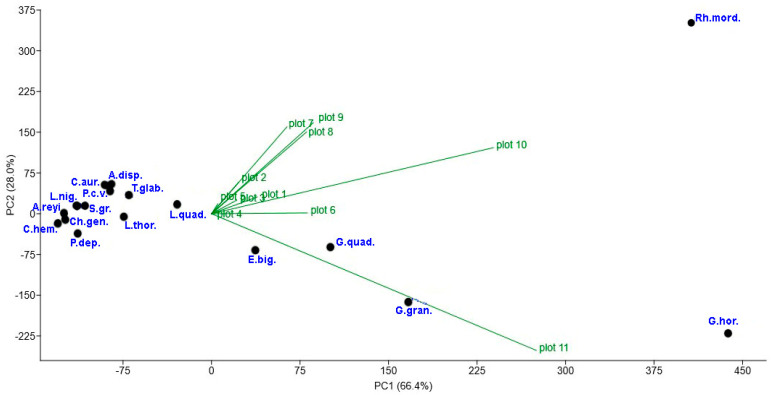
The diagram of the principal component analysis ordination of the selected Coleoptera species in the Republic of Mordovia (European Russia) based on the number of specimens collected on various plots. C.aur.—*Cetonia aurata*; P.c.v.—*Protaetia cuprea volhyniensis*; T.glab.—*Trogoderma glabrum*; C.hem.—*Carpophilus hemipterus*; E.big.—*Epuraea biguttata*; G.gran.—*Glischrochilus grandis*; G.hor.—*Glischrochilus hortensis*; G.quad.—*Glischrochilus quadripunctatus*; S.gr.—*Soronia grisea*; P.dep.—*Pediacus depressus*; Ch.gen.—*Chrysanthia geniculata*; A.reyi—*Anastrangalia reyi*; L.quad.—*Leptura quadrifasciata*; L.thor.—*Leptura thoracica*; L.nig.—*Lepturalia nigripes*; Rh.mord.—*Rhagium mordax*; A.disp.—*Anisandrus dispar*. A brief description of the plots1–11 is given in Table 1.

Figure 4 shows that the abundance of most of the 17 species did not depend on the plots. However, five species (*E. biguttata*, *G. grandis*, *G. hortensis*, *G. quadripunctatus*, and *Rh. mordax*) were exceptions. The abundance of four species, namely, *E. biguttata*, *G. grandis*, *G. hortensis*, and *G. quadripunctatus*, showed substantially higher numbers on plot 10 and particularly on plot 11. This information pertains to the results of the Shannon and Simpson index calculations on plots 10 and 11 (Table 2). The calculations demonstrated the dominance of one or more Coleoptera species. For instance, *Rh. mordax* and these species represented the majority of individuals (67.7%) on plot 11. Meanwhile, *Rh. mordax* was the most abundant species on plot 10 (Figure 4).

**Figure 5:**
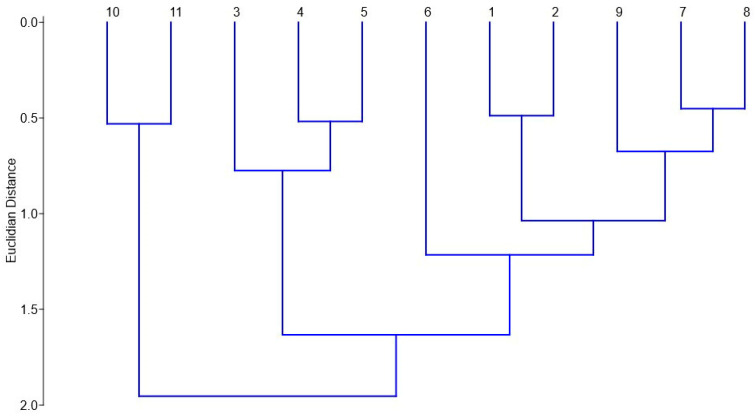
The similarity of Coleoptera species on 11 plots based on the Jaccard index.

The Jaccard index calculation revealed significant differences between the plots (Figure 5). The biodiversity of Coleoptera on plots 10 and 11, which were not affected by fires, was similar and significantly different from all other plots. Plots 3, 4, and 5, which burned in 2021, were grouped separately, while plot 6 occupied a distinct position. The fire margins were closely related with minimal differences in species composition.

## 4. Discussion

Our study investigated the effectiveness of using beer traps at low altitudes to study Coleoptera biodiversity in burned areas. The methodology has been extensively studied in open biotopes and forest edges [33,56,57] and can be applied to analyze biodiversity, faunal similarity, and other indicators. Our results indicate that beer traps located at low altitudes in open biotopes effectively attract Coleoptera and can profitably be used in other similar studies. The 2021 fires in the Mordovia State Nature Reserve destroyed dry trees, deadwood, and herbaceous vegetation in many areas, making the use of beer traps on tripods appropriate and effective.

The 2021 fires are considered catastrophic events due to their rapid spread over large areas during windy weather. These events can have devastating impacts on ecosystems, including the entomofauna. Such evaluations are objective and based on observable consequences. For instance, Duelli et al. [58] observed significant increases in insect species diversity and abundance in forests following windstorms. Similarly, Moretti et al. [59] and Bogusch et al. [60] found a similar effect among certain insect groups following significant fires in various European countries. However, severe fire impacts can significantly delay the recovery of insect fauna over large areas. In these cases, the entire ecosystem is disturbed, resulting in the death of plants across all forest layers, leading to a decrease in both biodiversity and abundance insects [61,62].

Small fires in disturbed forests allow more sunlight to reach the soil, promoting the growth of grasses, shrubs, and young trees. These conditions are more attractive to phytophagous, anthophilous, and other ecological groups of insects, which, in turn, become prey for predatory insects. Sunlight also promotes the reproduction and larval development of these insects on grasses, shrubs, and forest edges [14,57,63]. Low-intensity fires typically result in increases in species diversity and abundance. Small fires weaken deadwood, dry trees, and stumps, leading to the development of large numbers of saproxylic Coleoptera species [64,65,66].

In 2022, the traps recorded a 37% higher abundance of Coleoptera in the year following the fires compared to the second year (2023) after the fires. It is unclear whether this result is related to the duration of the experiment, as the traps were operated for 160 days in 2022 and 181 days in 2023. The reasons for this result are likely due to both unknown meteorological factors and the observed increase in abundance of many insect species after fires. These are the typical characteristics of mixed forests in the temperate zone. Forest plots 1 and 8 experienced fires in 2010 but not in 2021. The regeneration of birch and pine is slow, and there is a significant amount of dead wood, including dry dead trees resulting from the fires.

Plots 3, 4, and 5 had the lowest total abundance of Coleoptera. In addition to the fire of 2021, these suffered previous fires in 2010, which destroyed almost all deadwood, dry trees, herbaceous and shrub layers, and a young birch forest down to the roots in 2021. In the second year following the 2021 fires on plots 4, 5, and 6, there was a small increase in the number of Coleoptera species.

According to the Jacquard index the Coleoptera fauna showed the greatest similarity on plots 10 and 11, which formed a distinct clade that differed significantly from all others. Plots 1 and 2, plots 4 and 5, and plots 7 and 8 were also characterized by similar values. All of them were located close to each other or had similar conditions for beetles to live in. Plots 3, 4, and 5 also formed a distinct clade, while plot 6 was somewhat separated on the dendrogram. These results suggest that the faunas of the burned plots are similar to each other, and the beetle faunas of the control plots are more similar to one another than to the other plots.

Only 17 species of Coleoptera were found on all plots. However, the principal component analysis did not reveal any changes in the abundance of 12 out of these 17 species. That is, 12 species are quite widespread in biotopes and can inhabit both open ecosystems (burned plots) and forest ecosystems. Only five species showed certain trends in preference. For instance, *E. biguttata* can survive on rotting mushrooms, berries, fruits, and decaying liquids [67]. Adults of *G. grandis* and *G. hortensis* are commonly found on decaying tree sap from various trees, while their larvae develop on different decaying substrates [68,69]. *G. quadripunctatus* and *Rh. mordax* are commonly found in subcortical habitats of both coniferous and deciduous trees, where they feed on decaying tree sap from various tree species [70,71].

The study showed that the diversity and abundance of Coleoptera species were greater on unburned plots than on burned ones. Moreover, plots with one or more species dominance led to a decrease in the Shannon index and an increase in the Simpson index. These indicators changed inversely on plots without herbaceous vegetation, shrubs, deadwood, and dead trees. It is a well-established fact that fires on small plots promote subsequent infestation in neighboring unburned plots [61,62]. However, plots with highland fires may be adjacent to plots with lowland fires. In such cases, the fauna of completely burned plots takes longer to recover [29]. However, if all possible feeding objects for Coleoptera adults and larvae are completely destroyed by fire, recovery may take even longer. The relative number of saproxylic and anthophilic species varied on different plots, confirming this fact. The burned plots would be mainly colonized by saproxylic species that inhabit deadwood or remnants of dead trees. Following primary fires, these organics attract numerous saproxylic invertebrates [72,73]. However, a secondary fire in 2010 destroyed the maximum possible habitats with decaying organics. As a result, there was no significant increase in saproxylic abundance.

## 5. Conclusions

We surveyed 11 plots and collected 12,218 specimens of 194 Coleoptera species from 38 families over the course of two years. The families Nitidulidae, Cerambycidae, Elateridae, and Scarabaeidae were the most abundant across all plots. Cerambycidae, Elateridae, Nitidulidae, Staphylinidae, Coccinellidae, and Scarabaeidae were the families with the greatest species diversity. All plots had 17 species in common, while only 5 species showed a preference for specific plots. The unburned (control) plots yielded the highest abundance and species diversity. Meanwhile, plots that experienced fires in 2010 and 2021 had the lowest total Coleoptera abundance values. The fire in 2021 on these plots destroyed nearly all objects that could be used for the settlement, feeding, breeding, and sheltering of beetles. In the first year after the fire of 2021, the traps recorded a higher abundance of Coleoptera than in the subsequent year. The coleopteran fauna were most similar on the control and on nearby plots.

## Figures and Tables

**Table 1:** A brief description of the plots in the places of installation of traps.

Plots	Burned	Description
1	2010	In 2021, not exposed to fire. Some amount of dead wood, trees (*Pinus* and *Betula*). Dense undergrowth of birch. Shrubs are mainly represented by raspberries (*Rubus*). Grassy tier sparse. Some birch litter.
2	2010	In 2021, not exposed to fire. Located 10 m from edge of 2021 fire. Similar in all respects to plot 1.
3	2010 and 2021	In 2021, the territory was completely burned out. The deadwood and grassy tier are completely burnt out. There were rare dry bushes. It is located 10 m from the edge of the fire deep into the burnt territory (20 m from plot 2).
4	2010 and 2021	In 2021, the territory was completely burned out. It is located 1000 m from the edge of the fire deep into the burned area. The deadwood, shrubs, and grassy tier were completely burned out.
5	2010 and 2021	It is located 2000 m from the edge of the fire deep into the burned territory. Similar in all respects to plot 4.
6	2010 and 2021	It is located 1500 m from the edge of the fire deep into the burned territory. Lowland with water (wet biotope). In 2021, there was a low-intensity fire. Fallen trees and rare birch undergrowth, left over from the fires of 2010, have been preserved. The grassy tier almost completely burned out in 2021.
7	2010 and 2021	It is located 10 m from the edge of the fire deep into the burnt territory (20 m from plot 8). The deadwood, birch, shrubs, and grassy tier were partially burned out. At least half of the dead wood and a lot of dense dry birch undergrowth remained.
8	2010	It is located 10 m from the edge of the fire in 2021. A significant amount of large deadwood trees (pine and birch). Very dense undergrowth of birch and aspen. The grassy tier is sparse. The litter from the fall of hardwoods is small.
9	2010 and 2021	It is located 10 m from the edge of the fire deep into the burnt territory (20 m from plot 10). The deadwood, birch, shrubs, and grassy tier were partially burned out. At least half of the dead wood and dense dry birch undergrowth remain.
10	Not burned (control)	A plot of forest that has not been exposed to fire. It is located 10 m from the edge of the fires of 2010 and 2021. Old mixed forest of *Pinus sylvestris*, *Betula* with an admixture of *Tilia cordata*, *Sorbus aucuparia* (in the second tier). The litter is well-defined and powerful, and the grassy layer is sparse.
11	Not burned (control)	It is located 500 m from the edge of the fires. The old mixed forest is similar in configuration to plot 10. The litter is well-defined and powerful, and the grassy layer is sparse.

**Table 2:** The main parameters of Coleoptera individuals collected using beer traps on various plots (2022 and 2023).

Indicators	Plots										
	1	2	3	4	5	6	7	8	9	10	11
Number of families	21	20	15	18	12	21	24	20	22	24	23
Number of species (excluding unidentified ones)	72	65	49	53	53	66	81	78	77	82	80
Number of specimens	1293	910	555	646	457	1003	1165	1360	1198	1635	1996
Percentage of species that were saproxylic beetles (% of the total number of species on plots)	77.8	80.0	85.7	79.2	79.2	75.8	76.5	82.1	87.0	81.7	82.3
Percentage of species that were anthrophilic beetles (% of the total number of species on plots)	59.7	55.4	46.9	52.8	47.2	45.5	54.3	57.7	51.9	51.2	54.4
Margalef index	9.91	9.39	7.6	8.04	8.49	9.41	11.33	10.67	10.72	10.95	10.27
Shannon index	3.13	3.2	3.08	3.02	3.09	2.99	3.11	3.23	3.08	2.83	2.57
Simpson index	0.08	0.06	0.07	0.08	0.07	0.09	0.08	0.07	0.09	0.12	0.14

## Data Availability

All data are available from the authors upon request.

## Appendix A

**Table A1:** Species diversity of Coleoptera on plots during two years of study (S—saproxylic; A—anthophilous).

Species			Plots										
	S	A	1	2	3	4	5	6	7	8	9	10	11
**Carabidae**													
*Harpalus signaticornis* (Duftschmid, 1812)												1	
*Lebia marginata* (Geoffroy, 1785)												1	
*Tachyta nana* (Gyllenhal, 1810)	+								1		1		
**Histeridae**													
*Gnathoncus buyssoni* (Auzat, 1917)	+											1	3
*Margarinotus striola* (C.R. Sahlberg, 1819)	+											2	7
*Platysoma angustatum* (J.J. Hoffmann, 1803)	+		1										
*Platysoma elongatum* (Thunberg, 1787)	+												2
**Leiodidae**													
*Anisotoma axillaris* (Gyllenhal, 1810)	+								1				
**Staphylinidae**													
*Bolitochara pulchra* (Gravenhorst, 1806)	+										1		
*Carphacis striatus* (G.-A. Olivier, 1795)	+		1		1		1		1	2	3	5	51
*Deliphrum tectum* (Paykull, 1789)	+								1	1			
*Necrodes littoralis* (Linnaeus, 1758)						1	1	1		1			
*Nicrophorus investigator* (Zetterstedt, 1824)												2	
*Nicrophorus vespilloides* (Herbst, 1783)												2	
*Oiceoptoma thoracicum* (Linnaeus, 1758)									1	1	1		6
*Omalium rivulare* (Paykull, 1789)	+										1		1
*Phloeostiba plana* (Paykull, 1792)	+		1	1		1		1				3	9
*Phyllodrepa floralis* (Paykull, 1789)	+										1		
*Phyllodrepa melanocephala* (Fabricius, 1787)			1					1					
*Philonthus* sp.											2	1	8
*Quedius cruentus* (G.-A. Olivier, 1795)	+				1							1	2
*Quedius dilatatus* (Fabricius, 1787)	+											3	2
*Quedius scitus* (Gravenhorst, 1806)	+												1
*Staphylinidae* sp.			4	3				20	1	7	3	25	54
*Thamiaraea cinnamomea* (Gravenhorst, 1802)	+		1										1
**Lucanidae**													
*Platycerus caraboides* (Linnaeus, 1758)	+									1			1
**Scarabaeidae**													
*Cetonia aurata* (Linnaeus, 1758)	+	+	33	120	39	55	65	21	84	7	45	10	8
*Gnorimus variabilis* (Linnaeus, 1758)	+	+	5				1		6	12	1	2	2
*Oxythyrea funesta* (Poda von Neuhaus, 1761)		+	6	10	5	2	1		5	1	3		
*Protaetia cuprea volhyniensis* (Gory and Percheron, 1833)	+	+	50	53	29	30	22	2	66	32	48	18	13
*Protaetia fieberi* (Kraatz, 1880)	+	+	2	1	2		1		2	1	1		
*Protaetia marmorata* (Fabricus, 1792)	+	+	7	2		1	1	2	10	2	23	27	15
*Serica brunnea* (Linnaeus, 1758)			2							2			
*Trichius fasciatus* (Linnaeus, 1758)	+	+		3					2		1		5
**Scirtidae**													
*Contacyphon ochraceus* (Stephens, 1830)		+				1		2					1
*Contacyphon padi* (Linnaeus, 1758)		+	1										
*Contacyphon pubescens* (Fabricius, 1792)						1		2		1	1		1
*Contacyphon punctipennis* (Sharp, 1872)								21					
*Contacyphon variabilis* (Thunberg, 1787)		+	1					5	1	1			1
**Buprestidae**													
*Anthaxia quadripunctata* (Linnaeus, 1758)	+	+	3	4	1			1					
*Chalcophora mariana* (Linnaeus, 1758)	+								1				
*Chrysobothris chrysostigma* (Linnaeus, 1758)	+			1									
**Throscidae**													
*Trixagus* sp.	+				1						1	2	2
**Elateridae**													
*Agrypnus murinus* (Linnaeus, 1758)		+	34	16	19	13	20	9	18	57	12	2	
*Ampedus balteatus* (Linnaeus, 1758)	+	+	13	7	5	14	10		3	20	2	1	
*Ampedus cinnabarinus* (Eschscholtz, 1829)	+		135	64	97	110	38	196	154	137	167	46	
*Ampedus elongatulus* (Fabricius, 1787)	+		9	1		1		1	2	2			
*Ampedus nigrinus* (Herbst, 1784)	+								1				
*Ampedus nigroflavus* (Goeze, 1777)	+		4	1			1	5	2	6	1		
*Ampedus pomonae* (Stephens, 1830)	+		2	1	2	1		18	19	2		1	
*Ampedus pomorum* (Herbst, 1784)	+		26	1	3	1	1	40	20	80	3	8	
*Ampedus praeustus* (Fabricius, 1792)	+		20	5	9	16	7	12	39	47	9		
*Ampedus sanguineus* (Linnaeus, 1758)	+					7	8			1			
*Ampedus sanguinolentus* (Schrank, 1776)	+	+	2	2		1		10	4	2	6		
*Ampedus tristis* (Linnaeus, 1758)	+		1							1			
*Anostirus castaneus* (Linnaeus, 1758)	+				1		2						
*Cardiophorus ruficollis* (Linnaeus, 1758)	+		2		1	5	7	5			1		
*Dalopius marginatus* (Linnaeus, 1758)	+	+						5	1		2		
*Dicronychus equiseti* (Herbst, 1784)		+					2						
*Drapetes mordelloides* (Host, 1789)	+								1				
*Lacon lepidopterus* (Panzer, 1800)	+							1	1	7			
*Melanotus villosus* (Geoffroy, 1785)	+		2	1	3	82	41	2		3	3	2	3
*Prosternon tesselatum* (Linnaeus, 1758)		+	273	41	3				1	89	9	41	30
*Selatosomus aeneus* (Linnaeus, 1758)	+	+	2						2			1	
**Lycidae**													
*Lygistopterus sanguineus* (Linnaeus, 1758)	+	+		2					2	1	2		
**Cantharidae**													
*Cantharis livida* (Linnaeus, 1758)		+						1					
*Cantharis nigricans* (O.F. Müller, 1776)		+		1								2	1
*Cantharis obscura* (Linnaeus, 1758)		+										1	
*Cantharis pellucida* (Fabricius, 1792)		+		1								2	
*Cantharis rustica* (Fallén, 1807)		+						2					1
**Dermestidae**													
*Anthrenus museorum* (Linnaeus, 1761)		+							1				3
*Attagenus schaefferi* (Herbst, 1792)	+	+	3				2	2	2	11		22	3
*Ctesias serra* (Fabricius, 1792)	+	+							12	4	20	29	8
*Dermestus murinus* Linnaeus, 1758								1					
*Megatoma undata* (Linnaeus, 1758)	+	+	1	1							2	1	
*Orphilus niger* (P. Rossi, 1790)	+	+						2		2	4	2	1
*Trogoderma glabrum* (Herbst, 1783)	+	+	26	5	5	3	3	16	34	47	59	45	18
**Ptinidae**													
*Dorcatoma robusta* A. Strand, 1938	+							1					
*Hadrobregmus pertinax* (Linnaeus, 1758)	+											1	1
**Cleridae**													
*Thanasimus femoralis* (Zetterstedt, 1828)	+											1	
*Thanasimus formicarius* (Linnaeus, 1758)	+								1			2	
*Trichodes apiarius* (Linnaeus, 1758)		+	3	3		2	1		8	1	7		
**Melyridae**													
*Aplocnemus nigricornis* (Fabricius, 1792)	+	+										1	
*Dasytes niger* (Linnaeus, 1761)	+	+	3	11	1				1	4	2	2	6
*Malachius aeneus* (Linnaeus, 1758)	+	+	1										
*Malachius bipustulatus* (Linnaeus, 1758)	+	+								1			
**Monotomidae**													
*Rhizophagus fenestralis* (Linnaeus, 1758)	+							1		1	1	1	7
*Rhizophagus picipes* (G.-A. Olivier, 1790)	+												1
**Nitidulidae**													
*Carpophilus hemipterus* (Linnaeus, 1758)	+		6	10	8	5	11	20	5	2	7	7	1
*Carpophilus marginellus* (Motschulsky, 1858)	+			1			2	1	1	1	1	2	
*Carpophilus truncatus* (Murray, 1864)												1	
*Cryptarcha strigata* (Fabricius, 1787)	+		11	4	1		1	13	12	23	13	45	63
*Cryptarcha undata* (G.-A. Olivier, 1790)	+		2					1	1		1		4
*Cychramus luteus* (Fabricius, 1787)	+	+									1	6	3
*Epuraea biguttata* (Thunberg, 1784)	+		71	30	24	19	2	48	34	24	17	62	160
*Epuraea guttata* (G.-A. Olivier, 1811)	+							1			1	1	1
*Epuraea* sp.			16	5	4	2	4	14	5	8	10	48	76
*Glischrochilus grandis* (Tournier, 1872)	+	+	45	20	22	10	12	39	18	22	39	95	324
*Glischrochilus hortensis* (Geoffroy, 1785)	+		61	42	26	21	17	137	23	45	30	275	523
*Glischrochilus quadripunctatus* (Linnaeus, 1758)	+		16	31	29	37	23	54	26	34	48	120	196
*Glischrochilus quadrisignatus* (Say, 1835)	+				3			7	2	3	1	2	4
*Ipidia binotata* (Reitter, 1875)	+											1	
*Nitidula carnaria* (Schaller, 1783)					1		1						
*Omosita depressa* (Linnaeus, 1758)													1
*Omosita japonica* (Reitter, 1874)								1					
*Soronia grisea* (Linnaeus, 1758)	+		41	43	26	27	53	39	18	38	12	8	2
*Soronia punctatissima* (Illiger, 1794)	+		1				1				1		
**Cryptophagidae**													
*Cryptophagus* sp.						1							
**Silvanidae**													
*Ahasverus advena* (Waltl, 1834)						1		1					
**Cucujidae**													
*Cucujus haematodes* (Erichson, 1845)	+												1
*Pediacus depressus* (Herbst, 1797)	+		1	6	4	19	10	13	5	2	5	7	29
**Laemophloeidae**													
*Placonotus testaceus* (Fabricius, 1787)	+					1							
**Bothrideridae**													
*Bothrideres bipunctatus* (Gmelin, 1790)	+		1										
**Endomychidae**													
*Mycetina cruciata* (Schaller, 1783)	+										1		
**Coccinellidae**													
*Calvia decemguttata* (Linnaeus, 1767)		+	1										
*Calvia quatuordecimguttata* (Linnaeus, 1758)		+			1								
*Coccinella magnifica* (L. Redtenbacher, 1843)		+				1	1						
*Coccinella septempunctata* (Linnaeus, 1758)		+	2	5		1	1		2				
*Exochomus quadripustulatus* (Linnaeus, 1758)									1				
*Harmonia axyridis* (Pallas, 1773)							1						
*Hippodamia variegata* (Goeze, 1777)		+		1					1				
*Mysia oblongoguttata* (Linnaeus, 1758)								1					
*Oenopia conglobata* (Linnaeus, 1758)									1				
*Platynaspis luteorubra* (Goeze, 1777)		+							1				
*Propylea quatuordecimpunctata* (Linnaeus, 1758)		+										1	1
*Psyllobora vigintiduopunctata* (Linnaeus, 1758)		+		1								1	1
**Mycetophagidae**													
*Litargus connexus* (Geoffroy, 1785)	+						1					1	2
*Mycetophagus piceus* (Fabricius, 1777)	+			1								1	
*Typhaea stercorea* (Linnaeus, 1758)	+					1			1				
**Ciidae**													
*Cis comptus* (Gyllenhal, 1827)	+											1	
**Melandryidae**													
*Xylita laevigata* (Hellenius, 1786)	+										1	1	1
**Mordellidae**													
*Mordella* sp.	+	+		1									
*Mordellochroa abdominalis* (Fabricius, 1775)	+	+											1
**Tenebrionidae**													
*Bolitophagus reticulatus* (Linnaeus, 1767)	+			1							1		
*Hymenorus doublieri* Mulsant, 1852	+					1							
*Isomira murina* (Linnaeus, 1758)		+	3										
*Lagria hirta* (Linnaeus, 1758)		+	1					1	8	9		2	1
*Pseudocistela ceramboides* (Linnaeus, 1758)	+	+								1		1	
**Oedemeridae**													
*Chrysanthia geniculata* (W.L.E. Schmidt, 1846)	+	+	25	31	7	2	6	2	2	17	15	4	7
*Chrysanthia viridissima* (Linnaeus, 1758)	+	+	17	21	6	3	1	3		23	12	15	
*Oedemera femorata* (Scopoli, 1763)		+											1
**Pyrochroidae**													
*Schizotus pectinicornis* (Linnaeus, 1758)	+	+	1									1	
**Anthicidae**													
*Notoxus monoceros* (Linnaeus, 1761)		+	1	5	5				1	4	1		
**Scraptiidae**													
*Anaspis frontalis* (Linnaeus, 1758)	+	+	3					1	2	7	1	3	1
**Cerambycidae**													
*Anastrangalia reyi* (Heyden, 1889)	+	+	10	15	3	3	1	13	19	22	21	1	2
*Anastrangalia sanguinolenta* (Linnaeus, 1761)	+	+				2		2	2	2	11		1
*Arhopalus rusticus* (Linnaeus, 1758)	+						1						2
*Aromia moschata* (Linnaeus, 1758)	+	+			1				3	2			
*Dinoptera collaris* (Linnaeus, 1758)	+	+											1
*Judolia sexmaculata* (Linnaeus, 1758)	+	+				1		1	3	2		1	3
*Leptura aurulenta* (Fabricius, 1793)	+	+							1				
*Leptura quadrifasciata* (Linnaeus, 1758)	+	+	43	54	41	55	14	19	57	35	57	42	68
*Leptura thoracica* Creutzer, 1799	+	+	31	6	11	16	2	8	11	43	32	30	41
*Lepturalia nigripes* (De Geer, 1775)	+	+	49	64	13	23	6	5	7	32	26	7	3
*Lepturobosca virens* (Linnaeus, 1758)	+	+	5	8		3		1	1	1	14	3	1
*Molorchus minor* (Linnaeus, 1758)	+	+							1	1		1	1
*Necydalis major* Linnaeus, 1758	+	+	3			1		2	2		4	12	2
*Pachyta lamed* (Linnaeus, 1758)	+	+								1			
*Pachyta quadrimaculata* (Linnaeus, 1758)	+	+			1						1	4	2
*Phymatodes testaceus* (Linnaeus, 1758)	+	+		1								1	
*Phytoecia pustulata* (Schrank, 1776)												1	
*Plagionotus detritus* (Linnaeus, 1758)	+					1							
*Purpuricenus globulicollis* Dejean, 1839	+	+	2	1				2					
*Purpuricenus kaehleri* (Linnaeus, 1758)	+	+		1	3	2	2	4	5	1	2	1	
*Rhagium inquisitor* (Linnaeus, 1758)	+			1	2	3	1		18	14	35	33	11
*Rhagium mordax* (De Geer, 1775)	+	+	102	104	61	24	34	117	241	260	283	424	147
*Rhagium sycophanta* (Schrank, 1781)	+		2	3						1	1	4	
*Rutpela maculata* (Poda von Neuhaus, 1761)	+	+									1		1
*Stenocorus meridianus* (Linnaeus, 1758)	+	+	3	4		2				1	2	2	1
*Stenurella bifasciata* (O.F. Müller, 1776)	+	+									1		1
*Stenurella melanura* (Linnaeus, 1758)	+	+	1	1			1			7	4	5	11
*Stictoleptura maculicornis* (De Geer, 1775)	+	+			1	1				1			3
*Stictoleptura rubra* (Linnaeus, 1758)	+	+	3			2	1		2	1			
*Strangalia attenuata* (Linnaeus, 1758)	+	+							1	1	1		
*Xylotrechus antilope* (Schoenherr, 1817)	+		1										
*Xylotrechus capricornus* (Gebler, 1830)	+			1									
*Xylotrechus rusticus* (Linnaeus, 1758)	+		1	1	2			1	4		2	6	2
**Chrysomelidae**													
*Altica* sp.						1			3				
*Lilioceris merdigera* (Linnaeus, 1758)													1
*Phyllotreta atra* (Fabricius, 1775)								1	1				
*Plagiodera versicolora* (Laicharting, 1781)										1			
*Spermophagus sericeus* (Geoffroy, 1785)		+	6										
**Anthribidae**													
*Dissoleucas niveirostris* (Fabricius, 1798)	+									1			
*Platyrhinus resinosus* (Scopoli, 1763)	+				1								
*Tropideres albirostris* (Schaller, 1783)	+			1	2			2	3		2		
**Curculionidae**													
*Anisandrus dispar* (Fabricius, 1792)	+		23	16	9	4	5	17	91	63	26	24	5
*Brachyderes incanus* (Linnaeus, 1758)	+				1								
*Cryptorhynchus lapathi* (Linnaeus, 1758)	+									1			
*Hylastes opacus* (Erichson, 1836)	+			2									1
*Hylurgus ligniperda* (Fabricius, 1787)	+						3						
*Larinus obtusus* Gyllenhal, 1835											1		
*Lixus filiformis* (Fabricius, 1781)									1				
*Magdalis ruficornis* (Linnaeus, 1758)	+												1
*Notaris scirpi* (Fabricius, 1792)									1				
*Orchestes rusci* (Herbst, 1795)										1			
*Phyllobius pyri* (Linnaeus, 1758)			1	1			1						
*Rhinusa neta* (Germar, 1821)											1		
*Sibinia viscariae* (Linnaeus, 1761)				1									
*Sitona inops* Schoenherr, 1832								1					
*Sitona lineatus* (Linnaeus, 1758)							1						
*Strophosoma capitatum* (De Geer, 1775)				3	8	2			5	4	2	1	
